# Validity of self-reported receipt of iron supplements during pregnancy: implications for coverage measurement

**DOI:** 10.1186/s12884-019-2247-1

**Published:** 2019-04-02

**Authors:** Mufaro Kanyangarara, Joanne Katz, Melinda K. Munos, Subarna K. Khatry, Luke C. Mullany, Neff Walker

**Affiliations:** 1 Department of International Health, Johns Hopkins Bloomberg School of Public Health, Maryland, 615 North Wolfe Street, Baltimore, MD 21205 USA; 2 Nepal Nutrition Intervention Project – Sarlahi (NNIPS), Kathmandu, Nepal

**Keywords:** Antenatal care, Iron supplementation, Coverage, Validity, Low- and middle-income countries

## Abstract

**Background:**

Iron-deficiency anemia during pregnancy is an underlying cause of maternal deaths, and reducing risk through routine iron supplementation is a key component of antenatal care (ANC) programs in most low- and middle income countries. Supplementation coverage during pregnancy is estimated from maternal self-reports in population-based household surveys, yet recall bias and social desirability bias lead to errors of unknown magnitude.

**Methods:**

We linked data from household and health facility surveys from 16 countries to estimate input-adjusted coverage of iron supplementation during pregnancy. We assessed the validity of reported receipt of iron supplements in client exit interviews using direct observation as the gold standard across 9 countries with a recent Service Provision Assessment (SPA). Using a sample of 227 women who participated in the Nepal Oil Massage Study (NOMS), we also assessed the validity of self-reported receipt of iron folic acid (IFA) supplements. We used Poisson regression models to explore the association between client and health facility characteristics and agreement of self-reported receipt of iron supplements compared to direct observation.

**Results:**

Across the 16 countries, iron supplements were in supply at most of the 9215 sampled health facilities offering ANC services (91%). We estimated that between 48 and 93% of women attended at least one ANC visit at a health facility with iron supplements available. The specificity of recall of receipt of iron supplementation immediately following a visit was 79.3% and the sensitivity was 88.7% for the entire sample. Individual-level accuracy was high (Area under the curve > 0.7) and population bias low (0.75 < inflation factor < 1.25) across all countries. By contrast, in the NOMS sub-study, the accuracy of self-reported receipt of IFA supplements after 1–2 years was poor (sensitivity 86.1%, specificity 34.3%). Adjusted regression analyses indicated that older age and higher level of education were associated with poorer agreement between self-reports and direct observation.

**Conclusions:**

These findings suggest the need for caution when using self-reported measures with an extended recall period. Further validation studies using conditions similar to widely used population-based household surveys are warranted.

## Background

Anemia during pregnancy is a public health problem affecting 38% of pregnant women globally [1]. Iron deficiency is the most common cause of anemia and the underlying cause for an estimated 22% of maternal deaths [2]. Iron and folic acid (IFA) supplementation during pregnancy is a low-cost and effective method to reduce the burden of maternal anemia, sepsis, low birth weight, and preterm birth [3–5]. The World Health Organization (WHO) recommends daily supplementation with 30-60 mg of elemental iron and 0.4 mg of folic acid starting as early as possible in pregnancy [5]. Provision of iron supplements during pregnancy to prevent maternal anemia has been implemented extensively in antenatal care (ANC) programs across low- and middle-income countries (LMICs). Nevertheless, recent data from 36 LMICs suggest that less than one third (30%) of mothers consumed IFA supplements for 90 or more days during pregnancy [6].

Valid and reliable measurement of coverage of IFA supplementation during pregnancy is essential at global and national levels to guide policy, program planning and evaluation. Self-reported receipt and consumption of iron supplements during pregnancy is used to calculate population-level coverage of iron supplementation during pregnancy. A commonly reported indicator is the percentage of women with a birth in the 5 years preceding the survey who took iron-containing tablets or syrup for 90 days or more in their most recent pregnancy [7]. While definitions of indicators to track coverage of iron supplementation during pregnancy vary, the most common source of coverage data in LMICs is the Demographic and Health Surveys (DHS). The DHS collect data on the content of ANC received during the last pregnancy, including antenatal iron supplementation. Women of reproductive age (15–49 years) with a live birth in the previous 5 years are asked: “During this pregnancy, were you given or did you buy any iron tablets or syrup? During the whole pregnancy, for how many days did you take the tablets or syrup?” To aid recall, enumerators use visual aids with pictures of common iron supplements. An underlying assumption in these population-based surveys is that women can accurately report on the receipt and consumption of iron supplements for recall periods extending up to 5 years [8]. However, recall may be inaccurate if women do not know or remember receiving supplements or the number of days consumed. Women may also provide positive responses to IFA receipt and consumption if they perceive it is thought by others to be good for themselves or their fetuses, even if they did not consume supplements (social desirability bias). Factors that contribute to the accuracy of self-reported information may include demographic factors such as age, level of education and socioeconomic status, and survey-related factors such as the sensitivity of questions, the length of the recall period, and the timing (e.g. intrapartum period) [9–13]. Self-reporting on the timing and sequence of events is even more problematic especially among vulnerable populations and during the intrapartum period [14, 15]. The accuracy and reliability of self-reported data can impede the validity of inferences. Therefore, understanding the potential for errors can help the development of indicators, improve survey design and optimize data collection.

Despite the wide use of self-reported information on receipt and consumption of iron supplements during pregnancy, limited validation data exists [16]. A few studies have assessed the validity of maternal recall of the content of health services received at or around the time of birth; however, concerns remain about recall [9–11, 13, 17]. The objectives of this study were threefold (Table 1). First, we sought to estimate the coverage of antenatal iron supplementation in 16 LMICs by linking household and health facility survey data. Second, we assessed the validity of self-reported receipt of iron supplements using multi-country data from direct observations as the gold standard in a sample of pregnant women who were interviewed immediately following their consultation. We also examined the impact of the length of recall period on the validity of reported receipt of iron supplements using data from a trial in Nepal. Specifically, we directly compared retrospective (i.e. post-delivery) self-reported receipt of IFA supplements during pregnancy, with previously collected prospective documentation of receipt of supplement among a sample of pregnant women participating in a community-based randomized trial in Sarlahi District, Nepal. Lastly, we identified the characteristics of pregnant women in 9 countries who accurately reported receipt of iron supplements to understand variations in the accuracy of reporting.

**Table 1:** Study objectives and data sources

Study objective	Data source	Number of countries
Estimate the coverage of antenatal iron supplementation	Demographic and Health Surveys Service Provision Assessments (facility inventory) Service Availability and Readiness Assessments (facility inventory)	16
Assess the validity of self-reported receipt of iron supplements	Service Provision Assessment (client exit interview)	9
Examine the impact of the length of recall period on the validity of reported receipt of iron supplements	Nepal Oil Massage Study (NOMS) sub-sample	1
Identify the characteristics of pregnant women who accurately reported receipt of iron supplements	Service Provision Assessment (client exit interview)	9

## Methods

### Data sources

The study used household survey data from DHS and health facility survey data from Service Provision Assessments (SPA) and Service Availability and Readiness Assessments (SARA). The DHS are nationally representative household surveys that collect information on population and health indicators in LMICs. During interviews in sampled households, women 15–49 years of age are asked about ANC attendance and services received during their last pregnancy. The SPA and SARA are multi-country health facility surveys that evaluate the availability of and readiness to provide high quality health services at the national scale. Both tools include an inventory checklist of the availability of infrastructure, supplies, functional equipment, medicines and commodities for specific services including ANC. The SPA additionally include health provider interviews, observation of client provider consultations, and client exit interviews for specific services including ANC. For a systematic sample of ANC clients, enumerators directly observe and record whether management of care adhered to clinical guidelines. Following the client-provider consultations, structured exit interviews are conducted with the same ANC clients to collect information on basic sociodemographic factors, satisfaction with clinical practice and recall of clinical actions provided during visit. The interviews include a question about whether the ANC client was given or prescribed iron pills/syrup at the visit.

Countries with an available health facility survey (SPA or SARA) conducted in or after 2007 and a corresponding household survey conducted within 2 years of the index household survey were selected. Our analysis pooled data from 16 countries: Bangladesh, Benin, Burkina Faso, Democratic Republic of Congo, Haiti, Kenya, Malawi, Namibia, Nepal, Rwanda, Senegal, Sierra Leone, Tanzania, Togo, Uganda, and Zimbabwe. The assessment of validity of reported receipt of iron supplements was restricted to the 9 countries where observations of client provider consultations and client exit interviews for ANC services were conducted as part of the SPA.

We also used data collected as part of a sub-study of the Nepal Oil Massage Study (NOMS). NOMS was a cluster-randomized control trial assessing the neonatal mortality and morbidity impact of massaging newborns with sunflower seed oil instead of the traditionally used mustard seed oil in Sarlahi District, Nepal (ClinicalTrials.gov, NCT 01177111). The aim of the sub-study was to validate receipt of IFA supplements by comparing women’s retrospective self-report of receipt during pregnancy with prospectively collected study documentation recorded at the time of distribution by study workers. Specifically, a sample of 300 women who had participated in NOMS and who had been pregnant between January 31, 2016 and January 31, 2017 were randomly selected; 150 of the women were selected because there was documented receipt of IFA supplements from the Nepal Nutrition Intervention Project - Sarlahi (NNIPS), which conducted the NOMS trial. The other 150 women were selected because there was documentation that they had not received supplements through the study. Since the public health facilities in the area did not have IFA supplements during this period due to stockouts, it was unlikely these women would have received IFA supplements from the government health system. However, they may have bought or received supplements from the private sector. The final sample included 227 women who were successfully re-contacted and provided information on whether they received IFA tablets, capsules, or syrup during pregnancy using the Nepal DHS questions. Reasons for nonparticipation included: unavailability (*n* = 58), permanent migration outside the study area (*n* = 2), death (*n* = 4) and not contacted (*n* = 9). Trained study workers visited participants at home to conduct face-to-face follow-up interviews with standardized questionnaires. Questions included whether the respondent had received IFA supplements during pregnancy from NNIPS. Interviews were conducted in February of 2018; thus, the recall period was between one and 2 years.

### Analysis

#### Estimation of input-adjusted coverage

We defined the input-adjusted coverage of iron supplementation as the percentage of women who attended at least one ANC visit at a health facility with iron supplements available. To estimate input-adjusted coverage, we linked facility-level data on the availability of iron supplements from health facility surveys to data on the level of facility where ANC was accessed from the corresponding household surveys. Health facilities in each country were grouped into strata by facility type (hospital/health center/health post, etc) and managing authority (public/non-public) to obtain stratum-specific proportions of ANC facilities ‘ready’ to provide iron supplementation. From the household surveys, we computed ANC utilization by stratum, then weighted these estimates by the stratum-specific facility readiness measures to obtain coverage of iron supplementation. These coverage estimates represent the “input-adjusted coverage” or “availability coverage” [7, 18]. Linking methods have previously been used to estimate coverage of maternal, newborn and child health interventions not amenable to measurement using household surveys alone [19–21]. The linking method used in the present study has been described elsewhere [21]. Estimates of input-adjusted coverage obtained using linking were compared to the percentage of women with a live birth in the 3 years preceding the survey who received iron tablets or syrup during ANC derived from the DHS.

#### Validity of self-reported receipt of iron supplements

The validity of self-reported receipt of iron supplements was assessed by comparing self-reports from ANC clients during exit interviews with the “gold standard” of direct observation of clinical actions during the ANC consultation. Measures of validity calculated were sensitivity, specificity, area under the receiver operator characteristic curve (AUC) and the inflation factor (IF) [22]. The sensitivity was defined as the percentage of ANC clients who were given or prescribed iron supplements who correctly reported that clinical action during exit interviews. The specificity was defined as the percentage of ANC clients for which the clinical action was not observed who correctly reported the action not occurring. The AUC and IF were analyzed as measures of the accuracy of self-reporting at the individual and population-level, respectively. Based on prior validation studies, the acceptability criteria were defined as AUC > 0.7 and 0.75 < IF< 1.25 [12, 13, 23]. The same general approach to assessing validity was used for the NOMS data; however, validity relied on comparison of documented receipt of IFA supplements during scheduled contacts while participating in the NOMS trial and women’s subsequent recall of receipt of IFA supplements during the same pregnancy.

#### Factors associated with agreement

The outcome variable was agreement between self-reported receipt of iron supplements or a prescription for them and direct observation (yes/no). Because of the high prevalence of the outcome, we used a modified Poisson regression approach with robust error variance to describe the association between client and facility characteristics and agreement [24]. Potential covariates assessed included characteristics of ANC clients such as age (< 25, 25–34, ≥35 years), level of education (none, primary, secondary or higher), literacy (can read and write, cannot read and write), and gravidity (primigravida/multigravida); and characteristics of the clinical care and facility such as first ANC visit to facility (yes/no), facility nearest home (yes/no), type of facility (hospital/other), managing authority (public/non-public), fee for services (yes/no), and sex of provider seen (male/female). The choice of potential explanatory variables was based on availability in the client exit interview questionnaire. Models specified country-level fixed effects and adjusted for the complex sampling design. Unadjusted and adjusted risk ratios (RR) and corresponding 95% confidence intervals (CI) were calculated. A *p*-value less than 0.05 was considered statistically significant. All statistical analyses were performed using STATA 14 (StataCorp, College Station, TX).

## Results

### Estimation of input-adjusted coverage

**Fig. 1:**
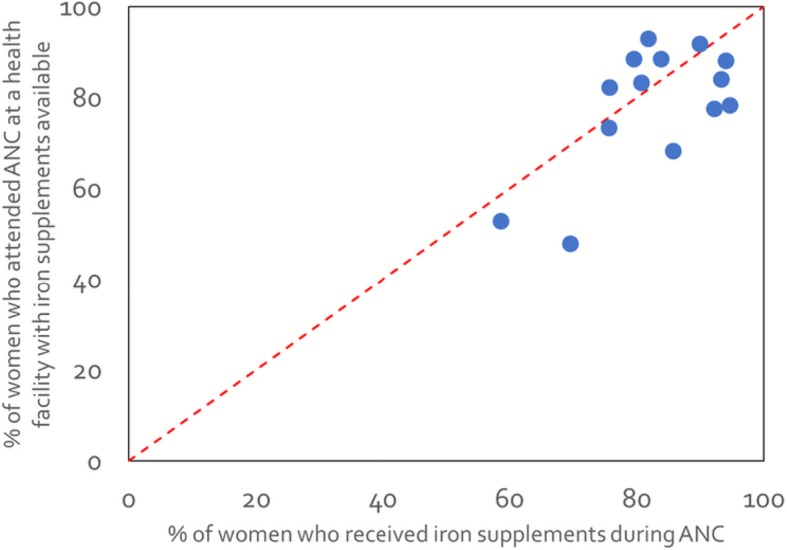
Comparisons of indicators of iron supplementation

Of 11,013 facilities sampled in the 16 countries between 2007 and 2016, 9215 reported offering ANC services and were included in the analysis (Table 2). About 9 in 10 ANC facilities had iron supplements available on the day of assessment (across country median: 91%). Availability of iron supplements at ANC facilities ranged from 52% in Kenya (2010) to 97% in Malawi (2013–14) and Benin (2013). Based on linking data on availability of iron supplements at ANC facilities from the SPA and SARA with data on where women accessed ANC from the DHS, we estimated that between 48 and 93% of women attended at least one ANC visit at a health facility with iron supplements available. Comparison of estimates of the percentage of women who attended ANC at a health facility with iron supplements available with the percentage of women who received iron tablets or syrup during ANC derived from the DHS indicated a linear association (Fig. 1). Spearman’s correlation coefficient was 0.50 and achieved borderline significance (*p* = 0.05).

**Table 2:** Health facility readiness and coverage of antenatal iron supplementation in 16 countries, 2007–2016

Country	Type of health facility survey	Year of health facility survey	Year of household survey^a^	ANC facilities sampled (n)	ANC facilities with iron supplements available (%)	Women who attended at least one ANC visit (%)^b^	Women attended ANC at a health facility with iron supplements available (%)
Bangladesh	SPA	2014	2014	1493	94	64	60
Benin	SARA	2013	2011–12	158	97	86	83
Burkina Faso	SARA	2012	2010	609	88	96	84
DRC	SARA	2014	2013–14	1149	60	89	53
Haiti	SPA	2013	2012	832	82	90	73
Kenya	SPA	2010	2008–09	561	52	92	48
Malawi	SPA	2013–14	2015–16	643	97	95	92
Namibia	SPA	2009	2006–07	303	94	94	89
Nepal	SPA	2015	2016	902	91	86	78
Rwanda	SPA	2007	2007–2008	432	78	97	75
Senegal	SPA	2016	2016	353	82	96	78
Sierra Leone	SARA	2013	2013	428	90	98	88
Tanzania	SPA	2014–15	2015–16	1031	95	98	93
Togo	SARA	2012	2013–14	92	93	73	68
Uganda	SPA	2007	2006	399	63	93	59
Zimbabwe	SARA	2014	2015	262	96	92	89
Median					91	92	78

*ANC* antenatal care, *DRC* Democratic Republic of Congo, *SPA* Service Provision Assessment, *SARA* Service availability and readiness assessment

^a^Demographic and Health Surveys (DHS) were used for all countries. ^b^Source: Corresponding Demographic and Health Survey

### Validity of self-reported receipt of iron supplements

Data from direct observations of ANC consultations and exit interviews conducted during the SPA were available for 13,880 clients seeking ANC at health facilities in 9 countries. The characteristics of ANC clients and facilities where they sought care are presented by country in (Table 3). Overall, most ANC clients were under 35 years of age (91%), had some primary education or higher (80%), and were able to read and write (63%). Furthermore, most ANC clients had a prior pregnancy (69%) and sought ANC at the public (76%) facility nearest their home (85%). Few ANC clients sought care at hospitals (28%) or health facilities that charged for ANC services (30%).

**Table 3:** Characteristics of ANC clients in 9 countries

	n	%
Client age (years)		
< 25	6655	49
25–34	5670	42
35+	1302	9
Client highest level of school attended		
None	2694	20
Primary	6792	50
Secondary or higher	4087	30
Client can read and write^a^	7116	63
Primigravida	4141	31
First visit to this facility for this pregnancy	5865	43
Facility nearest home	11,476	85
Hospital	3824	28
Public facility	10,254	76
Charged for services today	4019	30
Male provider	11,013	81
Country (year of survey)		
Haiti (2013)	1652	12
Kenya (2010)	1431	11
Malawi (2013–14)	2067	15
Namibia (2009)	857	6
Nepal (2015)	1542	11
Rwanda (2007)	734	5
Senegal (2016)	848	6
Tanzania (2014–15)	4073	30
Uganda (2007)	369	3

Data are weighted. ^a^Literacy was missing 17%, all other variables were missing < 5%

**Fig. 2:**
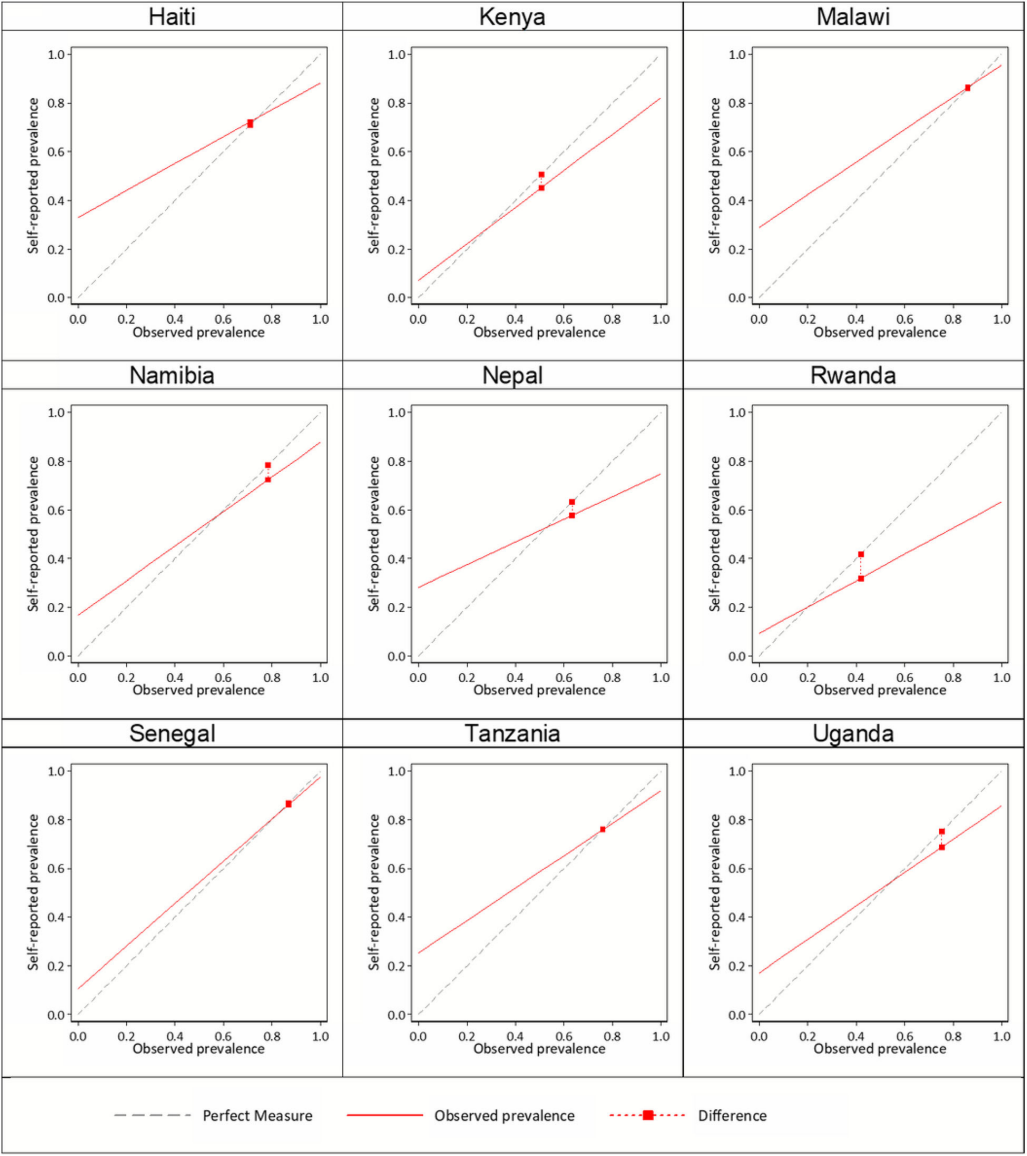
Observed versus self-reported receipt of iron supplements by country

During client exit interviews, 69.5% of ANC clients reported receiving iron supplements or a prescription for them during their visit (range: 31.9–86.3%) (Table 4). On the other hand, 71.8% of ANC clients were directly observed receiving iron supplements or a prescription for them (range: 41.8–86.8%). For the total sample, self-reported receipt of antenatal iron supplementation had a sensitivity of 88.7%, (range: 63.3–97.7%). The specificity of maternal recall ranged from 66.9 to 92.8% across countries, with a median of 79.3% for the total sample. Except for Kenya and Rwanda, sensitivity consistently exceeded specificity. In other words, the percentage of women who reported receiving supplements or a prescription when they had not (false positives) was higher than the percentage of women who reported not receiving iron supplements or a prescription yet were observed receiving them (false negatives). The lowest AUC was 0.73 (Nepal 2015) and the highest 0.93 (Senegal 2016). The IF was close to 1 for all countries indicating low population bias (range: 0.76–1.02; Fig. 2). All 9 countries met the acceptability criteria for individual-level classification (AUC > 0.7) and for valid approximation of population- level coverage (0.75 < IF< 1.25) (Fig. 2; Table 4). These findings suggest that women were able to accurately recall receiving iron supplements or a prescription for them immediately following an ANC visit.

**Table 4:** Validity assessment of self-reported receipt of iron-containing supplements in 9 countries, 2007–2016

Country	Year	Survey	Total number of ANC clients observed	Observation prevalence	Sensitivity	Specificity	Correctly classified	Self-reported prevalence	Area under the curve (AUC)	Inflation factor
Haiti	2013	SPA	1620	70.9	88.3	66.9	82.1	72.3	0.78	1.02
Kenya	2010	SPA	1409	50.6	82.1	92.8	87.4	45.1	0.87	0.89
Malawi	2013–14	SPA	2068	86.0	95.7	71.0	92.2	86.3	0.83	1.00
Namibia	2009	SPA	859	78.4	87.7	83.3	86.7	72.3	0.86	0.92
Nepal	2015	SPA	1565	63.3	74.7	71.8	73.6	57.6	0.73	0.91
Rwanda	2007	SPA	722	41.8	63.3	90.7	79.2	31.9	0.77	0.76
Senegal	2016	SPA	849	86.8	97.7	89.3	96.6	86.2	0.93	0.99
Tanzania	2014–15	SPA	4009	75.9	91.9	74.8	87.8	75.8	0.83	1.00
Uganda	2007	SPA	779	75.2	85.7	82.9	85.0	68.7	0.84	0.91
Total			13,880	71.8	88.7	79.3	86.0	69.5	0.84	0.97
Nepal	2016–17	NOMS	227	53.7	86.1	34.3	62.1	76.7	0.60	1.43

*ANC* antenatal care, *NOMS* Nepal Oil Massage Study, *SPA* Service Provision Assessment

Using the Nepal SPA 2015, the sensitivity of self-reported receipt of iron supplements was 74.7% and specificity was 71.8% (Table 4). Based on the AUC and IF, the acceptability criteria for accurate reporting was met. By contrast, the sensitivity based on NOMS sub-study data was higher (86.1%), and the specificity much lower (34.3%) than the validation results based on the SPA data. A higher percentage of women reported receiving IFA supplements when they had not (high false positive rate). The acceptability criteria for validity of self-reported receipt of IFA supplements was not met as the AUC was 0.60 and the IF was 1.43. These findings suggest poor accuracy of recall between one and 2 years.

### Factors associated with agreement

In the multivariate analyses, agreement between self-report and direct observation was associated with age, education level, whether this was the first ANC visit, and whether the health facility charged for ANC services (Table 5). Poor agreement was observed when the ANC client was 35 years of age or older (aRR: 0.96, 95% CI: 0.93–0.98), had a secondary education or higher (aRR: 0.97, 95% CI: 0.95–0.99), and was charged for ANC services received (aRR: 0.93, 95% CI: 0.93–0.96). On the other hand, visiting the facility for first time during the current pregnancy predicted good agreement (aRR: 1.07, 95% CI: 1.06–1.08).

**Table 5:** Factors associated with agreement between self-reports and direct observation of receipt of iron supplementation among ANC clients from 9 countries, 2007–2016

	Crude risk ratio	95% CI	Adjusted risk ratio	95% CI
Client age (years)				
< 25	Reference		Reference	
25–34	0.99	0.97–1.00	0.98	0.97–1.00
≥35	0.97	0.95–1.00	0.96	0.93–0.98
Client highest level of school attended				
None	Reference		Reference	
Primary	1.01	0.98–1.02	1.00	0.99–1.02
Secondary or higher	0.95	0.93–0.97	0.97	0.95–0.99
Primigravida	0.97	0.96–0.99		
First visit to this facility for this pregnancy	1.06	1.05–1.08	1.07	1.06–1.08
Facility nearest home	1.05	1.03–1.07		
Hospital	0.95	0.93–0.96		
Public facility	1.05	1.03–1.06		
Charged for services today	0.96	0.95–0.98	0.94	0.93–0.96
Male provider	1.03	1.01–1.05		
Country				
Nepal (2015)	Reference		Reference	
Haiti (2013)	1.10	1.06–1.15	1.12	1.08–1.16
Kenya (2010)	1.18	1.14–1.22	1.20	1.16–1.24
Malawi (2013–14)	1.24	1.20–1.29	1.22	1.18–1.26
Namibia (2009)	1.17	1.12–1.22	1.15	1.11–1.20
Rwanda (2007)	1.07	1.02–1.12	1.05	1.00–1.10
Senegal (2016)	1.30	1.26–1.35	1.35	1.30–1.39
Tanzania (2014–15)	1.18	1.15–1.22	1.16	1.13–1.20
Uganda (2007)	1.15	1.10–1.20	1.13	1.08–1.17

*ANC* antenatal care, *CI* confidence interval

## Discussion

Population-based coverage of antenatal IFA supplementation is a core indicator of progress towards the reduction of anemia and can be used to monitor and evaluate programs and policies for antenatal IFA supplementation. Household surveys have been widely used to track coverage of health interventions including antenatal IFA supplementation. Therefore, the validity of measures obtained from household surveys is important to assess as inaccurate recall has implications for program planning, monitoring country progress, and evaluating programs [11]. This study sought to estimate the input-adjusted coverage of antenatal iron supplementation. The availability of iron supplements at health facilities was widespread across the 16 countries studied (across country median: 93%), reflecting the adequacy of iron supplement procurement and supply. By linking household and health facility survey data, we estimated that most women attended ANC at a health facility that had iron supplements available (across country median: 78%). Our input-adjusted coverage estimates are much higher than recent estimates based on population-based surveys that suggest that a third of women consume IFA supplements for 90 days or more during pregnancy [6]. While our methods and indicator definitions vary, these findings suggest that women may not be receiving 90 days’ worth of supplements or may not be taking all the supplements they receive.

This study also assessed the criterion validity of self-reported receipt of iron supplements in a sample of pregnant women seeking ANC at health facilities across 9 countries. The sensitivity of self-report compared to direct observation ranged from 63.3 to 97.7%, and the specificity from 66.9 to 92.8%. Although there was heterogeneity in accuracy across countries, AUC values (> 0.7) were high and inflation factors close to 1 for both the entire sample and individual country samples. Based on these findings suggest that self-reports in client exit interviews provided acceptable measures of receipt of iron supplements among women attending ANC. Client exit interviews were conducted immediately following the ANC visits, therefore it is possible that as the recall period lengthens, women may recall receipt of iron supplements less well. An important aspect of this study was the examination of the validity of retrospective self-report for assessing the receipt of IFA supplements. A sample of 227 Nepalese women who participated in the Nepal Oil Massage Study were followed up 1–2 years after they received supplements from the program. Recall accuracy was poor; sensitivity was 86.1% and specificity was 34.3%. Such low levels of specificity could arise from social desirability bias or poor recall of receipt of supplements from NNIPS. Although there were major stock-outs of IFA supplements at public facilities during the time of the study, women may have received supplements from pharmacies or private facilities and failed to remember whether the supplements came directly from NNIPS or somewhere else. Because of the differences in methodology between these two validation datasets we should use caution in comparing the results; however, our finding suggest that recall accuracy may deteriorate between the ANC visit and 1–2 years after delivery.

In our assessment of the factors associated with agreement between self-reports and direct observation, we found that older age (≥35 years) and higher educational attainment (secondary or higher) were independently associated with reduced agreement. Higher levels of reporting agreement were observed among women for which this was the first visit to the facility for the current pregnancy. Given the wording of the question in the exit interviews, it is possible that women with more than one ANC visit misunderstood the question or misclassified ANC receipt at the current visit versus the previous visit.

The strengths of this study include the use of a large sample of ANC clients and health facilities across several countries, the linkage of client exit interview responses to direct observation data, and the availability of client and facility-level data to examine factors affecting recall accuracy. There are several limitations worth noting. First, the definition of input-adjusted coverage used – the percentage of women who attended ANC at a health facility with iron supplements available - lacked information on actual receipt and consumption during pregnancy. Similarly, the measure used in the validation analysis reflects whether women were given or prescribed iron supplements but does not reflect who took the supplements or how many were consumed. Due to limitations in the wording of the questions in the exit interviews and observation protocols in the SPA, no attempt was made to distinguish between receipt of iron supplements and receipt of a prescription. Second, direct observations were considered the “gold standard,” but records may be incomplete or inaccurately reflect a patient encounter. For example, all clinical actions completed during a consultation may not be documented if an ANC client saw multiple providers during the same visit. In this case, the lack of documentation by a third party observer does not guarantee that the clinical action did not take place. Teasing out the degree of reporting inaccuracy attributable to measurement error is challenging. Third, caution is necessary when considering the generalizability of findings to other settings. The SPA interviews a sample of women seeking care at sampled ANC facilities, therefore findings reflect reporting accuracy of women who sought facility-based care. However, it is unlikely that women not accessing ANC received and consumed IFA. Lastly, the study design and available data did not allow the exact replication of DHS field conditions where women would be asked to report about iron supplements during pregnancy that occurred up to 5 years preceding the survey. Exit interviews in the SPA occurred immediately after visit and the questions used were different from the DHS questions. The NOMS data in which women were followed up 1–2 years after is more representative of DHS conditions. These limitations draw attention to the need to improve the design of validation studies to reflect actual survey conditions. Additionally, supplementing validation results with qualitative research could enhance the development and validity of appropriate indicators. A qualitative lens could be used to explore how women understand questions, the phrasing of specific questions, or even ways to improve recall.

## Conclusions

While maternal recall of receipt of iron supplements seemed to be valid immediately following the visit, data from NOMS trial in Nepal support the caution expressed by other studies about the use of long recall periods. Accurate nutrition surveillance is important to track progress towards the targets to reduce anemia. With increased calls for quality health services, these findings have implications for the evaluation of quality of services [21, 25]. Further research is needed to support the development and validation of nutrition indicators to ensure that robust findings inform policy decisions and the allocation of resources for public health.

## Abbreviations

ANC: Antenatal care
AUC: Area under the curve
CI: Confidence interval
DHS: Demographic and Health Surveys
IF: Inflation factor
IFA: Iron and folic acid
LMICs: Low- and middle-income countries
NNIPS: Nepal Nutrition Intervention Project - Sarlahi
NOMS: Nepal Oil Massage Study
RR: Risk ratio
SARA: Service Availability and Readiness Assessment
SPA: Service Provision Assessment
WHO: World Health Organization
